# Structure and enzymology of glutaminase S482C and H461L variants associated with excess brain glutamate and neurological disease

**DOI:** 10.1016/j.jbc.2026.113091

**Published:** 2026-04-27

**Authors:** Cléa S. Crane, Thora K. McIssac, Shawn K. Milano, Richard A. Cerione, Scott M. Ulrich

**Affiliations:** 1 Department of Chemistry and Chemical Biology, Cornell University, Ithaca, New York, USA; 2 Department of Molecular Medicine, Cornell University, Ithaca, New York, USA; 3 Department of Chemistry, Ithaca College, Ithaca, New York, USA

**Keywords:** glutaminase, disease-associated variant, excitotoxicity, glutamate, neurotransmission, enzyme mechanism, protein structure

## Abstract

Glutaminase variants associated with neurological diseaseGlutaminase (GLS) catalyzes the hydrolysis of glutamine to produce glutamate, the brain’s principal excitatory neurotransmitter. Two *de novo* gain-of-function mutations in GLS, S482C and H461L, were recently identified in patients with developmental delay, epilepsy, and infantile cataract. These patients exhibited high glutamate and low glutamine concentrations in the brain, suggesting that the GLS variants have abnormal enzymology. Here, we examined the enzymatic properties of the mutant enzymes and found that they no longer require the anionic activator phosphate to stimulate enzymatic activity or induce filament formation. The mutant enzymes also exhibit a total (S482C) or partial (H461L) loss of glutamate product inhibition, lifting this restriction on glutamate accumulation. Structural analysis of the S482C variant shows the mutation shifts the key catalytic residue Y466 into its catalytically active configuration and disrupts a key hydrogen bond between Y466 and the glutamate product, explaining how the S482C variant has enzymatic activity in the absence of phosphate and is insensitive to glutamate product inhibition. These results shed new light on the mechanism of phosphate activation and glutamate product inhibition of GLS and show that loss of these enzymatic properties disrupts glutamate homeostasis in the brain and causes neurological disease.

Mammalian glutaminases (EC 3.5.1.2) are tetrameric mitochondrial enzymes that catalyze the hydrolysis of glutamine to glutamate (1, 2). There are two glutaminase genes in humans, *GLS* and *GLS2*. Glutaminase (*GLS*) encodes kidney-type glutaminase (KGA) and a C-terminal splice variant glutaminase C (GAC), while *GLS2* encodes liver-type glutaminase (LGA). *GLS* isoforms KGA and GAC (collectively referred to here as GLS) are highly expressed in the brain and are the main producers of the excitatory neurotransmitter glutamate. GLS and the opposing enzyme glutamine synthetase compose the glutamine-glutamate cycle that maintains glutamate homeostasis in the brain (3). Presynaptic neurons release glutamate to activate glutamate receptors on postsynaptic neurons. Glutamate is cleared from the synaptic cleft into astrocytes by high-affinity transporters, where it is converted to glutamine by glutamine synthetase. Glutamine is then shuttled from astrocytes back into neurons where it is hydrolyzed to glutamate by GLS, completing the cycle (4, 5).

Aberrant GLS activity disrupts glutamate homeostasis in the brain and causes neurological disease. Loss-of-function GLS mutations cause ataxia, neonatal seizures, cerebral edema, and structural brain abnormalities (6, 7). Elevated GLS activity in the brain causes excess brain glutamate and excitotoxicity (8, 9), a pathology of glutamatergic neurons that contributes to many neurological diseases including Alzheimer's disease (10), amyotrophic lateral sclerosis (11), HIV-associated neurocognitive disorder (12), and epilepsy (13). Similarly, transgenic mice engineered to overexpress the GAC isoform of GLS in the brain have excess brain glutamate and display learning defects and synaptic dysfunction (14).

Recent reports identified *de novo* gain-of-function GLS mutations in two patients with unexplained neurological disease. One patient carried a S482C mutation and exhibited profound developmental delay and infantile cataract (15). The second patient carried a H461L mutation and exhibited moderate developmental delay and epilepsy (16). Both patients were found to have low glutamine and high glutamate levels in the brain, the likely cause of their disease (17).

The reports that identified the S482C and H461L mutations and described their clinical effects proposed that the excess brain glutamate observed in the patients was due to hyperactivity of the GLS variants, but their enzymology was not studied in detail. The S482C and H461L mutations reside in the catalytic domain of GLS, which is identical in the KGA and GAC isoforms. The X-ray crystal structure of apo WT GAC shows that S482 is adjacent to the active site and contacts catalytic residue Y466, whereas H461 is a surface residue that contacts its twin on an adjoining subunit of the GLS tetramer (Fig. 1, *A*–*C*) (18). Sequence alignment shows that S482 is strictly conserved across the glutaminase superfamily while H461 shows some variation (Fig. S1).

**Figure 1:**
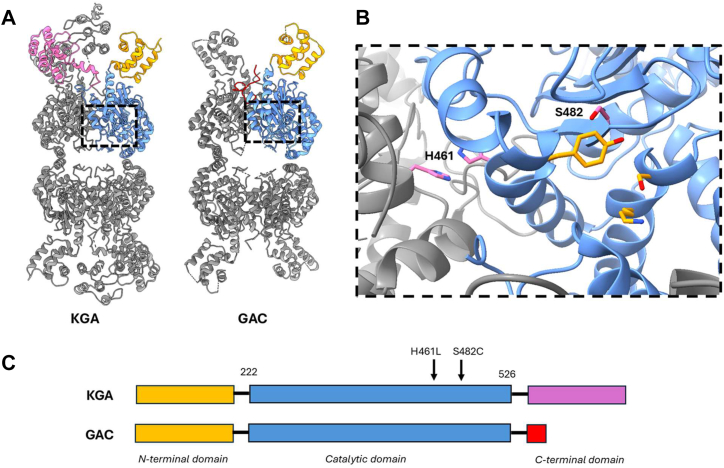
**Structure of the GLS isoforms and location of the disease-associated mutations.***A*, structures of the GLS isoforms KGA and GAC. The isoforms have identical N-terminal (*yellow*) and catalytic (*blue*) domains. The C-terminal domains differ in both length and sequence (GAC = *red*; KGA = *magenta*). *B*, detailed view of the GLS active site showing the S482 and H461 residues mutated in the patients (*pink*) as well as catalytic triad residues S286, K289, and Y466 (*orange*). S482 contacts the edge of catalytic residue Y466; H461 is at the monomer-monomer interface where it contacts its twin across the interface. *C*, domain structure of KGA and GAC showing the positions of the disease-associated mutations in the shared *blue* catalytic domain. Images generated from PDB IDs 5UQE (KGA) and 3UNW (GAC). GAC, glutaminase C; GLS, glutaminase; KGA, kidney-type glutaminase; PDB, Protein Data Bank.

Here, we sought to characterize the structure and enzymology of the S482C and H461L GLS variants. Understanding how the S482C and H461L mutations alter the biochemical and regulatory properties of GLS could shed new light on GLS enzymology, explain the excess glutamate observed in the patients, and identify the features of GLS that are essential to maintain glutamate homeostasis in the brain.

## Results

### S482C and H461L GLS variants do not require phosphate for enzymatic activity and are resistant to glutamate product inhibition

**Figure 2:**
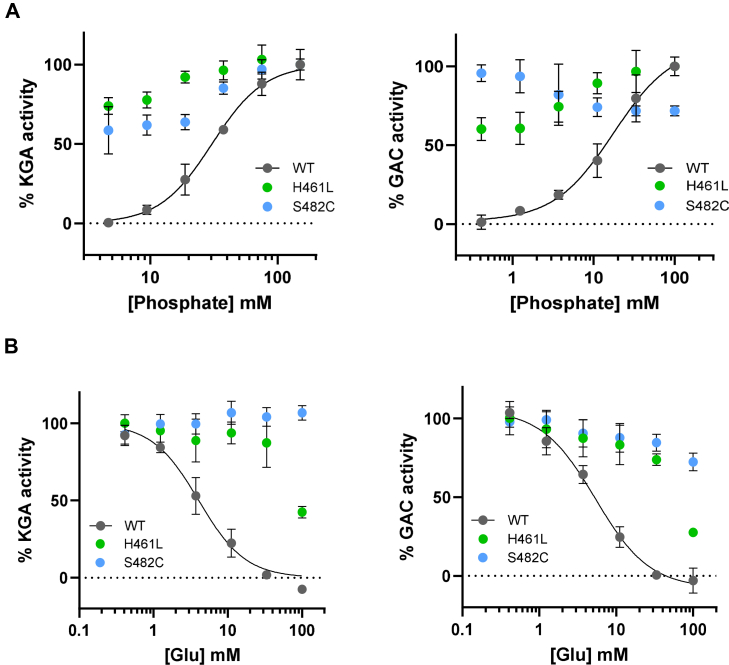
**Phosphate activation and glutamate product inhibition of WT GLS isoforms and the S482C and H461L variants.***A*, plots of the enzymatic activity of WT KGA and GAC and the S482C and H461L variants with increasing phosphate concentration, normalized to the maximal signal. *B*, plots of the enzymatic activity of WT KGA and GAC and the S482C and H461L variants with increasing glutamate concentration. The phosphate and glutamine concentrations were fixed at 15 mM each. Data are mean ± S.D., n = 3. GAC, glutaminase C; GLS, glutaminase; KGA, kidney-type glutaminase.

Phosphate is an essential anionic activator of GLS enzymes (19, 20). We measured the effect of phosphate on the activity of wild-type (WT) GLS isoforms KGA and GAC and the S482C and H461L variants of each. Similar to previous studies (19, 21, 22, 23) we found that WT KGA and GAC show very low activity in the absence of phosphate and are strongly activated by phosphate addition, with EC_50_ values of 30 ± 4 mM (KGA) and 16 ± 2 mM (GAC). Conversely, the S482C and H461L variants of KGA and GAC are active enzymes in the absence of phosphate, and the addition of phosphate only slightly affects their activity (Fig. 2*A*). High concentrations of phosphate modestly enhance the activity of the mutant enzymes, except for S482C GAC, which surprisingly shows slightly reduced activity.

The phosphate concentration in mitochondria is 15 mM (24, 25), close to the EC_50_ value of phosphate activation for GAC and half the EC_50_ value for KGA. At this phosphate concentration, WT GLS enzymes are partially activated, and their activity would robustly respond to changes in phosphate levels. In contrast, the S482C and H461L variants are active in the absence of phosphate and only modestly respond to changes in phosphate concentration.

GLS enzymes exhibit product inhibition by glutamate, which binds the active site and shows competitive binding with both glutamine substrate and the phosphate activator (1, 22, 26). We measured glutamate product inhibition of WT KGA and GAC and the S482C and H461L variants at 15 mM glutamine and 15 mM phosphate, equal to its concentration in mitochondria. Under these conditions, we found that WT KGA and GAC are inhibited by glutamate with IC_50_ values of 4.1 ± 0.5 mM (KGA) and 5.3 ± 0.7 mM (GAC), whereas the variants showed a complete (S482C) and partial (H461L) loss of glutamate product inhibition (Fig. 2*B*).

The glutamate concentration in neuronal mitochondria is reported to be 10 mM (27, 28, 29), twice the IC_50_ value for glutamate product inhibition of WT KGA and GAC, and sufficient to significantly inhibit the WT enzymes. Conversely, the GLS variants are insensitive (S482C) or resistant (H461L) to glutamate product inhibition. These findings suggest that glutamate product inhibition of WT GLS may well be an important regulator of brain glutamate levels, and that loss of product inhibition in the GLS variants contributes to the excess brain glutamate observed in the patients.

### The S482C and H461L mutations have modest effects on enzyme kinetics

**Figure 3:**
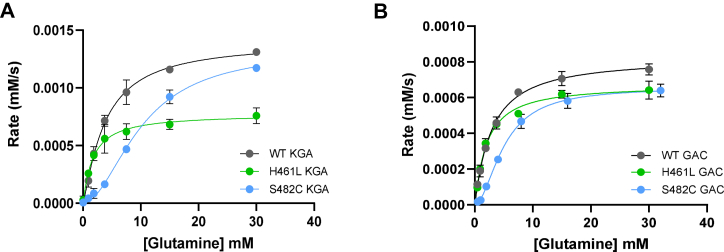
**Glutamine substrate concentration vs reaction rate plots.***A*, WT KGA and the S482C and H461L variants. *B*, WT GAC and the S482C and H461L variants. All reactions were carried out in the presence of 50 mM phosphate. Data are mean ± S.D., n = 3. GAC, glutaminase C; KGA, kidney-type glutaminase.

We measured the k_cat_ and K_0.5_ values of WT KGA and GAC and the S482C and H461L variants to determine if the variants have enhanced enzymatic capacity relative to the WT enzymes. We performed these measurements at 50 mM phosphate for all enzymes, which is twice the EC_50_ value for KGA activation and 3-fold higher than the EC_50_ value for GAC activation. Under these conditions, we found that the S482C and H461L variants of KGA and GAC have comparable kinetic parameters to the corresponding phosphate-activated WT enzymes (Fig. 3 and Table 1) (30). Therefore, the excess glutamate observed in the patients is not due to increased catalytic capacity of the S482C and H461L variants, but is instead due to the loss of phosphate activation and/or glutamate product inhibition.

**Table 1:** Kinetic parameters of WT KGA and GAC and the S482C and H461L variants

Enzyme	Kcat (s-1)	K0.5 glutamine (mM)	Hill slope	EC50 phosphate (mM)	IC50 glutamate (mM)
WT KGA	7.0 ± 0.5	3.8 ± 0.4	1.2 ± 0.1	30 ± 4	4.1 ± 0.5
S482C KGA	6.6 ± 0.5	10.1 ± 0.7	1.9 ± 0.1	-	-
H461L KGA	3.9 ± 0.8	1.7 ± 0.3	1.1 ± 0.2	-	-
WT GAC	16 ± 1	2.9 ± 0.3	1.1 ± 0.1	16 ± 2	5.3 ± 0.7
S482C GAC	13.1 ± 0.9	5.1 ± 0.3	1.9 ± 0.1	-	-
H461L GAC	13.4 ± 0.8	2.0 ± 0.2	1.1 ± 0.1	-	-

GAC, glutaminase C; KGA, kidney-type glutaminase.

Data are best-fit values ± SEM.

Surprisingly, the S482C variants of KGA and GAC exhibit sigmoidal kinetics, indicating a shift to positive substrate cooperativity (Fig. 3 and Table 1). The LGA enzyme encoded by *GLS2* also shows positive substrate cooperativity and lacks product inhibition by glutamate (1, 31). The catalytic domains of LGA and the GLS isoforms are ∼80% identical; however, the molecular basis for their distinct enzymatic properties remains unclear (2, 32). The observation that the S482C mutation in KGA and GAC results in LGA-like enzymatic properties could help address this question.

### The S482C and H461L variants constitutively assemble into filaments

Glutaminase enzymes are among a growing number of metabolic enzymes that reversibly assemble into filament-like structures (33, 34, 35). GLS filaments are the active form of the enzyme; phosphate induces filament formation while glutamate causes the filaments to disassemble into tetramers (36, 37, 38, 39). Since we observed that the S482C and H461L variants do not require phosphate for enzymatic activity and are resistant to glutamate product inhibition, we suspected that the regulation of filament formation by the variants would also be altered. Indeed, a recent report on GLS filamentation showed that S482C GAC formed filaments in the absence of phosphate and a cell-permeable glutamate precursor failed to disassemble S482C GAC filaments in cells (36).

**Figure 4:**
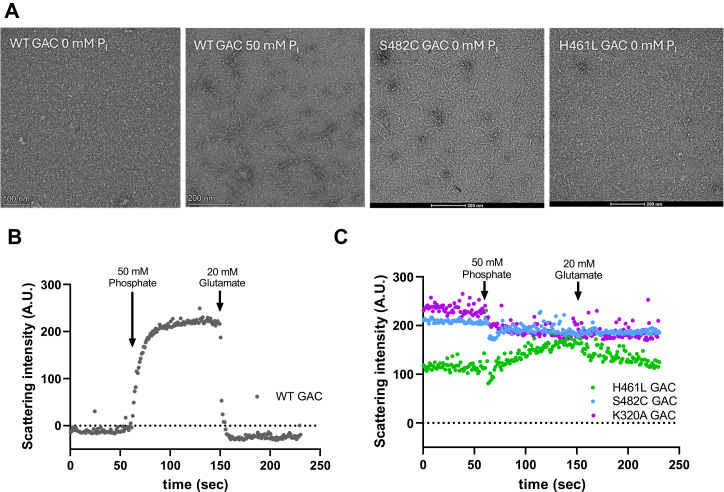
**Filament formation by WT GAC and the H461L and S482C variants.***A*, negative-stain electron microscopy images of WT GAC in the absence and presence of phosphate as well as the S482C and H461L variants in the absence of phosphate. Below each panel is the scale bar for that image. *B*, right-angle light scattering (RALS) assay of WT GAC showing an increase in light scattering upon addition of phosphate and a return to basal level of scattering upon addition of glutamate. *C*, RALS assay of the H461L, S482C, and K320A GAC variants showing no change (S482C and K320A) or a modest change (H461L) in light scattering upon addition of phosphate and glutamate. GAC, glutaminase C.

We evaluated filament formation by WT GAC and the S482C and H461L variants using negative-stain electron microscopy. The data confirm that WT GAC requires phosphate to assemble into filaments. Conversely, we found that S482C and H461L GAC both assemble into filaments in the absence of phosphate, although H461L filaments appear shorter than those of the S482C variant (Fig. 4*A*). We also measured filament formation of WT GAC and the S482C and H461L variants using right-angle light scattering, which allows the dynamics of filament assembly and disassembly to be monitored in real time (38, 40). We confirmed that phosphate promotes filament formation of WT GAC, indicated by an increase in light scattering upon phosphate addition. Subsequent addition of glutamate caused WT GAC filaments to disassociate, shown by a return to the basal level of light scattering (Fig. 4*B*). The K320A GAC variant is known to form filaments in the absence of phosphate (41) that are not disassembled by glutamate (36). We found that the intensity of light scattering by K320A GAC did not change upon phosphate or glutamate addition, consistent with constitutive filamentation (Fig. 4*C*). The S482C GAC variant also showed no change in scattering intensity upon addition of phosphate, consistent with filament formation in the absence of phosphate that we observed by electron microscopy. The scattering intensity of S482C GAC did not decrease upon glutamate addition, indicating its filaments resist disassembly by glutamate. The H461L GAC variant showed a slight increase in light scattering upon phosphate addition which was reversed by subsequent addition of glutamate, suggesting the small H461L GAC filaments observed by electron microscopy can be lengthened or enhanced by phosphate. The finding that the S482C and H461L GAC variants are constitutive filaments that do not respond (S482C) or weakly respond (H461L) to phosphate and glutamate aligns with our observation that these variants are active enzymes in the absence of phosphate and are insensitive (S482C) or resistant (H461L) to glutamate product inhibition.

We then tested whether the S482C and H461L GAC variants are sensitive to the GLS inhibitor CB-839. CB-839 is an anticancer clinical candidate that targets GAC, which is frequently overexpressed in cancer cells and is a key part of the metabolic reprogramming that supports tumor growth (42, 43). CB-839 binds at the dimer-dimer interface of GLS, which is remote from the S482C and H461L mutations (Fig. S2*A*). CB-839 binding locks GLS into inactive tetramers (30), and conditions that promote filament formation such as high concentrations of phosphate and the K320A mutation cause resistance to CB-839 (41, 44, 45). As such, CB-839 sensitivity can be used as a proxy measurement for GLS filament formation. We found that CB-839 potently inhibited WT GAC (IC_50_ = 58 ± 4 nM) while K320A GAC was insensitive to it, as expected (Fig. S2*B*). The S482C variant was also resistant to CB-839, whereas the H461L variant was weakly inhibited (IC_50_ = 350 ± 30 nM). These results further support the view that S482C and H461L GAC are constitutive filaments and that the H461L filaments are shorter and/or weaker than those of the S482C variant.

### X-ray crystal structure of S482C GAC

**Figure 5:**
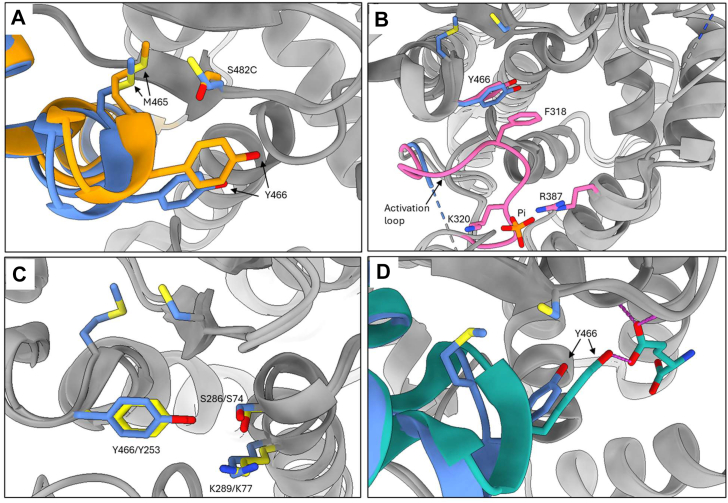
**X-ray crystal structure of the S482C GAC variant.***A*, superimposed structures of inactive apo WT GAC (*orange*) and active S482C GAC (*blue*). S482 of WT GAC contacts the catalytic residue Y466. Cysteine 482 of GAC S482C is rotated away from Y466 to pack against M465, causing a small displacement of the helix-turn-helix motif and shifting Y466 to a “down and in” configuration. *B*, superimposed structures of active phosphate-bound WT GAC (*pink*) and active S482C GAC (*blue*) show the same Y466 position. Phosphate promotes this conformation by ordering the activation loop which promotes a pi-stacking interaction between F318 and Y466. *C*, superimposed structures of *Bacillus subtilis* glutaminase YbgJ (*yellow*) and S482C GAC (*blue*). Shown are the Ser-Lys-Tyr catalytic triad of each enzyme, indicating that the position of Y466 in S482C GAC is the catalytically competent configuration. *D*, superimposed structures of active S482C GAC (*blue*) and inhibited glutamate-bound WT GAC (*teal*). The Y466 hydroxyl group makes a weak hydrogen bond to the glutamate side chain. The S482C mutation shifts Y466 out of hydrogen bond range and out of alignment with the glutamate side chain. Images generated from PDB IDs 9PIA (this study, S482C GAC); 3VOY (apo WT GAC); 8IMA (phosphate-bound WT GAC); 3UNW (glutamate-bound WT GAC); 1MKI (*B. subtilis* YbgJ) superimposed using the USCF ChimeraX Needleman–Wunsch tool for best aligning chains (52). GAC, glutaminase C; PDB, Protein Data Bank.

We solved the X-ray crystal structure of S482C GAC to understand how the mutation causes the loss of phosphate activation and glutamate product inhibition. Overall, the structures of inactive apo WT GAC and the S482C variant are very similar; however, we found significant differences at the site of the S482C mutation and at Y466, a key catalytic residue. In the apo WT GAC structure, the S482 hydroxyl group contacts the edge of the Y466 ring. The structure of the S482C GAC variant shows the mutant cysteine residue rotates away from Y466 to pack against the neighboring hydrophobic residue M465, both of which reside in the turn of a short helix-turn-helix motif. The packing of the mutant cysteine against M465 induces a small dip in this elbow-like motif which repositions Y466 (Fig. 5*A*).

The repositioning of Y466 by the S482C mutation explains how the S482C variant is active in the absence of phosphate. Phosphate binds WT GLS at the dimer-dimer interface using K320 and R387 as well as Y394′ and K398′ from the subunit across the interface (37, 39). K320 is part of the activation loop (L^316^RFNKLF^322^), which is disordered in the inactive apo WT enzyme. Phosphate binding causes the activation loop to become ordered, which initiates a pi-stacking interaction between activation loop residue F318 and catalytic residue Y466. This interaction shifts Y466 into its catalytically active configuration (37, 38, 39). This mechanism is supported by the observation that the F318S mutant, which cannot engage in the pi-stacking interaction with Y466, is inactive and does not respond to phosphate (37). Superimposing the structures of active phosphate-bound WT GAC and S482C GAC shows that the position of Y466 in the two structures is identical (Fig. 5*B*). The activation loop remains disordered in the S482C GAC structure, indicating that phosphate binding to the WT enzyme and the S482C mutation each promote the same catalytically active configuration of Y466 but in mechanistically distinct ways.

Comparing the structures of S482C GAC and *Bacillus subtilis* glutaminase YbgJ supports the idea that the position of Y466 in the S482C GAC structure is the catalytically active conformation. The catalytic domains of bacterial and mammalian glutaminases share a beta-lactamase fold and use a common catalytic triad consisting of S286, K289, and Y466 in GAC and S74, K77, and Y253 in YbgJ (46). YbgJ is active in the absence of phosphate, so its catalytic triad is likely in the catalytically active configuration. The position of the catalytic triad in S482C GAC and YbgJ are identical, suggesting that the catalytic triad residues of S482C GAC are also in their catalytically active conformation (Fig. 5*C*).

The repositioning of Y466 by the S482C mutation can also account for the loss of glutamate product inhibition by the mutant enzyme. The structure of glutamate bound to the active site of WT GAC shows the hydroxyl group of Y466 makes a weak hydrogen bond to the glutamate side-chain carboxylate, with a donor-acceptor distance of 3.3 Å (30). Superimposing the structures of glutamate-bound WT GAC and the S482C variant shows that the S482C mutation shifts Y466 out of hydrogen-bond range with glutamate (donor-acceptor distance of 3.9 Å) and moves the donor/acceptor groups out of alignment (Fig. 5*D*). This explains the S482C GAC mutant enzyme’s lower affinity for glutamate and loss of product inhibition.

These results show that there are two key positions of Y466. One position is catalytically competent and has a low affinity for glutamate, as seen in the structures of phosphate-bound WT GAC, the S482C GAC variant, and the bacterial glutaminase YbgJ. The second position is catalytically inactive and has a higher affinity for glutamate, as seen in the structures of apo- and glutamate-bound WT GAC.

## Discussion

In this study, we examined the structure and enzymology of two GLS variants identified in patients with elevated brain glutamate levels, neurological disease, and developmental delay. Our finding that the S482C and H461L GLS variants form catalytically active filaments in the absence of phosphate and resist glutamate product inhibition can account for the excess brain glutamate observed in the patients. GLS localizes to mitochondria where the phosphate concentration is 15 mM (24, 25), sufficient to partially activate WT GLS. Reports have shown that BCH domain family members BNIP-H/Caytaxin (47) and BMCC1S (48) transport the KGA isoform of GLS to the cytoplasm, where the phosphate concentration is significantly lower (24). Overexpression of BNIP-H/Caytaxin in 293T cells suppressed glutamate levels (47), consistent with deactivation of KGA by movement to a low phosphate environment. The S482C and H461L GLS variants are active in the absence of phosphate and therefore exhibit higher enzymatic activity than WT GLS under the low-phosphate conditions of the cytoplasm, contributing to the excess brain glutamate observed in patients. The glutamate concentration in neuronal mitochondria (as well as whole brain tissue) is 10 mM (27, 28, 29), sufficient to inhibit WT GLS and suppress further accumulation of glutamate. The loss of glutamate product inhibition by the S482C and H461L variants removes this restriction and likely also contributes to the excess brain glutamate observed in the patients. These results show that phosphate activation and glutamate product inhibition are essential properties of GLS to maintain glutamate homeostasis in the brain.

The structure of the S482C GAC variant reveals a mechanism for its enzymatic activity in the absence of phosphate and loss of glutamate product inhibition. Phosphate binds WT GLS at the dimer-dimer interface using residues of the activation loop, which becomes ordered upon phosphate binding. Ordering of the activation loop initiates a pi-stacking interaction between loop residue F318 and catalytic residue Y466. This interaction shifts Y466 into its catalytically competent position relative to the remaining catalytic residues S286 and K289 (37, 38, 39). The S482C variant uses a novel mechanism to achieve this catalytically competent active site configuration. The activation loop remains disordered in S482C GAC; instead, the mutant cysteine residue rotates to form a new hydrophobic contact with M465, inducing a small shift in the helix-turn-helix motif M465 resides in. This shift moves neighboring catalytic residue Y466 into the same catalytically active position caused by phosphate binding to WT GLS, explaining the activity of the S482C variant in the absence of phosphate. Phosphate binds GLS at a site that is somewhat distant from both the S482C and H461L mutations. Accordingly, although these variants do not require phosphate for activity, phosphate can still bind and exert a modest effect.

Glutamate product inhibition of GLS is partly mediated by a weak hydrogen bond between the glutamate side chain and the Y466 hydroxyl group (30). The position of Y466 induced by both phosphate binding to WT GLS and the S482C mutation moves Y466 out of hydrogen bond range and out of alignment with glutamate, explaining how phosphate suppresses glutamate product inhibition of WT GLS (26) as well as the loss of product inhibition by the S482C variant.

Some important questions remain regarding these disease-associated GLS variants. The effects of the H461L mutation on enzyme properties are similar to those of S482C, but the underlying mechanism is unclear due to the lack of structural data. The proximity of H461L to Y466 suggests it may exert its effects by repositioning Y466 analogous to the S482C mutation. A second unresolved question concerns the composition of the GLS variants *in vivo*. Because patients harboring the S482C and H461L mutations are heterozygous, hybrid WT/mutant GLS tetramers and/or filaments may form. Such heteromeric species may exhibit distinct biochemical properties from the homomeric enzymes characterized in this study.

In summary, patients carrying the S482C and H461L GLS variants exhibit elevated levels of brain glutamate and neurological disease. Our findings demonstrate that these mutations disrupt phosphate activation and glutamate product inhibition of GLS enzymatic activity. The structure of S482C GAC highlights the central role of catalytic residue Y466 in mediating these regulatory properties of GLS, which are essential for maintaining glutamate homeostasis in the brain.

## Experimental procedures

### Recombinant glutaminase expression and purification

An N-terminal 6His-tagged form of human KGA (UniProt ID: O94925–1) without the mitochondrial localization sequence (residues 1–71) was cloned into the pET28a plasmid. An N-terminal 6His-tagged form of human GAC (UniProt ID: O94925-3) without the mitochondrial localization sequence (residues 1–71) was cloned into the pQE80L plasmid (21). Site-directed mutagenesis was performed using Phusion DNA polymerase (New England Biolabs). The primers (5′–3′) used to introduce the mutations in GAC and KGA were as follows:

S482C: CTT CCT GCA AAA TGT GGA GTT GCT GGG (forward); CCC AGC AAC TCC ACA TTT TGC AGG AAG (reverse)

H461L: TTG AGT TTG ATG CTT TCC TGT GGC ATG (forward); CAT GCC ACA GGA AAG CAT CAA ACT CAA (reverse)

K320A: CTA AGA TTC AAC GCA CTA TTT TTG AAT (forward); ATT CAA AAA TAG TGC GTT GAA TCT TAG (reverse)

Expression and purification of glutaminase enzymes was carried out by transforming the constructs described above into *Escherichia coli* BL21 (DE3) competent cells (New England Biolabs), which were then grown in LB media overnight with 50 μg/ml kanamycin (KGA) or 50 μg/ml ampicillin (GAC). The starter cultures were used to inoculate 6 L of terrific broth (1:100 dilution) with the same antibiotic concentrations and shaken at 37 °C, 180 rpm for 3 to 4 h until the *A*_600_ reached between 0.6 and 0.8. The flasks were chilled at 4 °C for 1 h before induction with 100 μM IPTG and shaken at room temperature at 180 rpm for 16 h. Cells were collected by centrifugation at 5000*g* for 10 min and frozen. Frozen cell pellets were resuspended in 150 ml lysis buffer (50 mM Tris–HCl pH 8.5, 500 mM NaCl, and 10% glycerol) supplemented with protease inhibitor cocktail (Roche) then lysed by sonication. The mixture was clarified by ultracentrifugation (40,000*g*) for 45 min. The supernatant was then loaded onto Co^2+^ charged TALON resin (GoldBio), previously equilibrated with wash buffer (50 mM Tris–HCl pH 8.5, 10 mM NaCl, and 10 mM imidazole). The protein that bound to the column was washed with wash buffer (150 ml) and eluted with wash buffer supplemented with 320 mM imidazole. Further purification was performed by anion exchange chromatography using HiTrap Q HP column (Cytiva) and size-exclusion chromatography using Superdex 200 pg 16/600 column (GE HealthCare). Proteins were kept in 20 mM Tris–HCl pH 8.5, 150 mM NaCl, snap-frozen in liquid nitrogen, and stored at −80 °C. Protein concentrations were determined by absorbance at 280 nm using extinction coefficients calculated using the Expasy ProtParam tool.

### Glutaminase enzyme assays

Glutaminase was diluted in glutaminase buffer (65 mM Tris acetate pH 8.6, 0.2 mM EDTA) to a final concentration of 50 nM (GAC) or 200 nM (KGA) for each type of assay described below.

#### Michaelis–Menten kinetics

For the measurement of Michaelis –Menten kinetics, 80 μl of the enzyme mixture was added to 96 well plates. The glutaminase reaction was initiated by the addition of 20 μl of a solution of glutamine (150, 75, 37.5, 18.75, 9.37, and 4.69 mM) and K_2_HPO_4_ (250 mM) in glutaminase buffer, mixed by gently pipetting up and down and incubated at room temperature for 600 s.

#### Phosphate stimulation

The assay for phosphate stimulation was similar except the reaction was initiated by the addition of 20 μl of a solution of glutamine (100 mM) and K_2_HPO_4_ (500, 250, 125, 62.5, 31.2, 15.6, 7.8, and 0 mM) in glutaminase buffer, mixed by gently pipetting up and down and incubated at room temperature for 600 s.

#### CB-839 inhibition

The assay for CB-839 inhibition was similar except the enzyme mixture was incubated with 1.0 μl of a dimethyl sulfoxide solution of CB-839 and mixed by gently pipetting up and down. The reaction was initiated by the addition of 20 μl of a solution of glutamine (100 mM) and K_2_HPO_4_ (500 mM) in glutaminase buffer and incubated at room temperature for 600 s.

In the assays described above, the glutaminase reactions were quenched by the addition of 10 μl of cold HCl (3 M). An aliquot (10 μl) of each quenched glutaminase reaction was added to 190 μl of a glutamate dehydrogenase reaction, which consisted of Tris–HCl (100 mM, pH 9.4), NAD+ (2 mM), glutamate dehydrogenase (2 μl of a 50% glycerol solution, ≥35 units/mg protein), and hydrazine (1 μl) then incubated at room temperature for 40 min. The absorbance increase at 340 nm was measured and converted to glutamate concentrations using the extinction coefficient for NADH (6220 M^−1^ cm^−1^).

#### Glutamate inhibition

The assay to measure glutamate product inhibition used a smaller initial enzyme volume (70 μl) added to 96 well plates to which was added a solution of glutamate (10 μl) in glutaminase assay buffer (1000 mM, 333 mM, 111 mM, 37.0 mM, 12.3 mM, 4.1 mM, and 0 mM). The reaction was initiated by the addition of 20 μl of a solution of glutamine (75 mM) and K_2_HPO_4_ (75 mM) in glutaminase buffer, mixed by gently pipetting up and down and incubated at room temperature for 600 s. The reactions were quenched by addition of 10 μl of HCl (3 M). An aliquot (10 μl) of each quenched glutaminase reaction was added to 190 μl of a glutamate dehydrogenase reaction configured to measure the ammonia product which consisted of Tris–HCl (100 mM, pH 9.4), NADH (0.23 mM), α-ketoglutarate (3.4 mM), and glutamate dehydrogenase (2 μl of a 50% glycerol solution, ≥35 units/mg protein) then incubated at room temperature for 40 min. The decrease in absorbance at 340 nm was measured and converted to ammonia concentrations using the extinction coefficient for NADH (6220 M^−1^ cm^−1^). Discussion of the coupled GDH assay configured to detect the ammonia product of GLS and validation of this assay to measure glutamate product inhibition of GLS are described in the Supporting information (Fig. S3).

In the assays described above, the negative controls were quenched with HCl prior to initiating the reaction by addition of glutamine or a glutamine/phosphate mixture. *A*_340_ changes between negative controls and active enzyme were typically 0.2 to 0.4, which is within the linear range of the assay. Kinetic parameters, EC_50_ and IC_50_ values were determined by using the appropriate curve fitting equation in the GraphPad Prism software (GraphPad Software).

### Negative stain electron microscopy

Formvar/carbon film 200 mesh copper grids (Electron Microscopy Sciences, EMS) were plasma cleaned by using PELCO easiGlow system (TED PELLA). Glutaminase was dissolved in 20 mM Tris pH 8.5, 120 mM NaCl to a final concentration of 1.0 μM and inorganic phosphate K_2_HPO_4_ was added to a final concentration of 50 mM as indicated. Ten microliters of the mixture were applied onto the grids for a 60 s incubation and the excess protein solution was blotted with filter paper. Ten microliters of 2% uranyl acetate was applied to the grid for 30 s followed by blotting two consecutive times. The grids were air dried for 5 min and visualized by a Thermo Fisher Scientific F200Ci electron microscope at 120 keV.

### X-ray crystallography

Human GAC (S482C) was concentrated to 5 mg/ml using an Amicon ultrafiltration device (10 KDa cutoff; Millipore). Crystals were grown at 20 °C using the hanging drop vapor diffusion technique (2 μl of protein solution and 2 μl of reservoir solution), with the reservoir containing 10% PEG 6000 (w/v), 1 M LiCl, and 0.1 M Tris, pH 8.5. Crystals formed after 5 days. Glycerol was used as the cryoprotectant prior to plunge freezing. The diffraction data were collected at cryogenic temperature (100 K) at the Cornell High Energy Synchrotron Source (MacCHESS). The monomer extracted from the apo human WT GAC structure (Protein Data Bank ID: 5D3O) was used as the search model for molecular replacement. The data reduction was performed with HKL2000 prior to phasing and refinement using Phenix and Coot (49, 50, 51). The statistics of data collection and structure refinement are summarized in Table S1. The structure is deposited in the Protein Data Bank under PDB ID 9PIA.

### Right-angle light scattering

Buffer (20 mM Tris pH 8.5, 150 mM NaCl) filtered through a 0.2 micron filter was added to a 1.2 ml cuvette then inserted into a Varian Cary Eclipse fluorimeter and stirred with a magnetic stir bar at 25 °C and the instrument was zeroed. Frozen glutaminase aliquots were thawed on ice, centrifuged at 14k relative centrifugal force for 10 min to clear protein aggregates, then added to the cuvette to a final concentration of 2 μM. The scattering intensity was recorded using excitation and emission wavelengths of 340 nm (5 nm bandpass) for 300 s, during which time 20 μl of K_2_HPO_4_ (2.5 M) and 20 μl glutamate (1.0 M) dissolved in the same buffer were added at 60 s and 150 s, respectively.

## Data availability

Coordinates for S482C GAC have been deposited in the RCSB Protein Data Bank (www.rcsb.org) with accession code 9PIA. All other data are contained within the manuscript and the Supporting Information.

## Supporting information

This article contains supporting information (53).

## Conflict of interest

The authors declare that they have no conflicts of interest with the contents of this article.
