# Supplementary material for: Structure and enzymology of glutaminase S482C and H461L variants associated with excess brain glutamate and neurological disease
Source: J Biol Chem. 2026 Apr 27;302(6):113091. doi: 10.1016/j.jbc.2026.113091 (PMC13218152; doi:10.1016/j.jbc.2026.113091)
Supplement: Supporting Figures S1–S3 and Table S1 [file mmc1.docx]

**Supporting Information**

Structure and enzymology of glutaminase S482C and H461L variants associated with excess brain glutamate and neurological disease

Cléa S. Crane^1^, Thora K. McIssac^1^, Shawn K. Milano^1,2^, Richard A. Cerione^1,2^*, Scott M. Ulrich^3^

*^1^ Department of Chemistry and Chemical Biology, Cornell University, Ithaca, NY 14853, USA*

*^2^ Department of Molecular Medicine, Cornell University, Ithaca, NY 14853, USA*

*^3^ Department of Chemistry, Ithaca College, Ithaca, NY 14850, USA*

*Corresponding author: email: [rac1@cornell.edu](mailto:rac1@cornell.edu); phone: (607) 253-3888

**Supporting Information Materials:**

**Figure S1.** Sequence alignment of the catalytic domain of glutaminase enzymes.

**Figure S2.** S482C and H461L mutations confer resistance to GLS inhibitor CB-839.

**Figure S3.** Validation of the GDH coupled assay used to measure glutamate product inhibition of GLS.

**Table S1.** Crystallographic Data Collection and Model Refinement Statistics.

**Figure S1.** **Sequence alignment of the catalytic domain of glutaminase enzymes.** Human GAC (the catalytic domain of KGA is identical to GAC), human LGA, and glutaminases from several model organisms. Amino acid numbering is for human GAC. The conserved catalytic residues S286, K289, and Y466 are marked with green arrows; the sites of the S482C and H461L mutations are marked with light blue arrows.


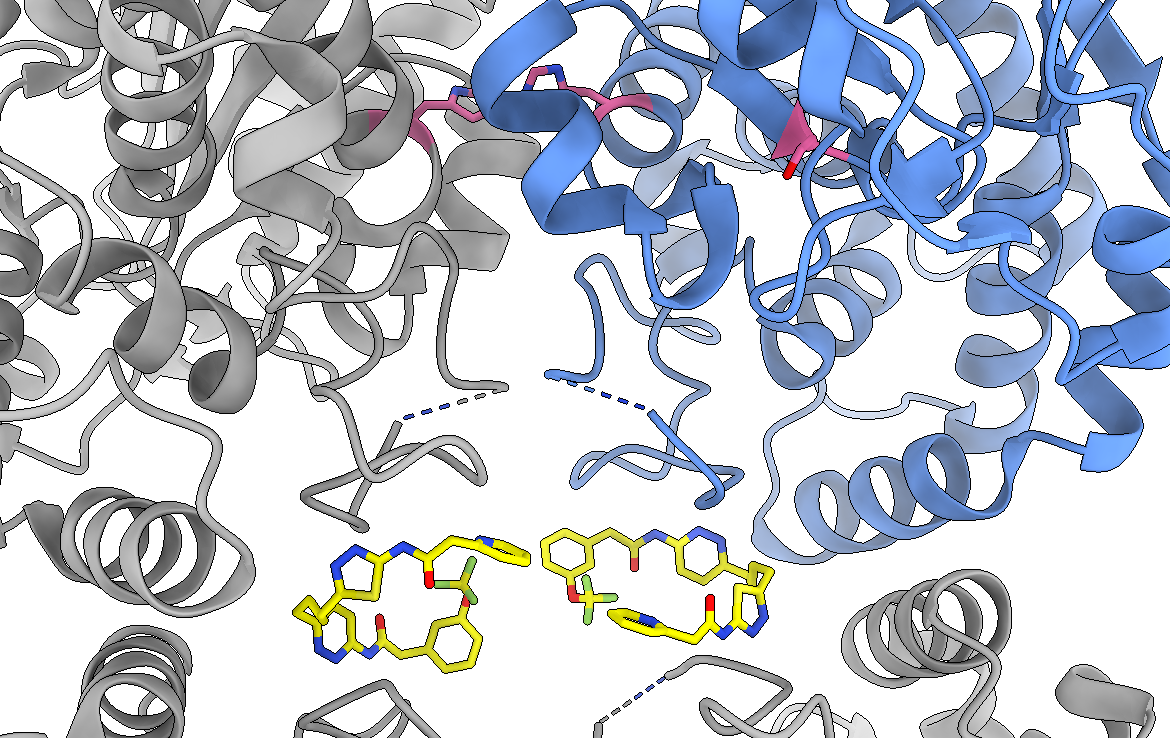


**B**

**A**

**Figure S2. S482C and H461L mutations confer resistance to GLS inhibitor CB-839. A)** CB-839 (yellow) binds GLS at the dimer-dimer interface (one GLS monomer is colored blue). The sites of the S482C and H461L mutations are shown in pink. Image generated from PDB ID 5JYO. **B)** Dose-response curves of the GLS inhibitor CB-839 on the enzymatic activity of WT GAC and the H461L, S482C, and K320A GAC mutants. *Data are mean ± S.D., n = 3.*

**A**

**B**

**C**

**Figure S3**: **Validation of the GDH coupled assay used to measure glutamate product inhibition of GLS**. **A)** The GDH coupled assay used to measure glutamate product inhibition of GLS was configured in the reductive amination direction to detect GLS-produced ammonia with concomitant consumption of NADH. **B)** The equilibrium position of the GDH reaction strongly favors the reductive amination direction.^53^ Glutamate added as an inhibitor to the GLS reaction could affect the GDH coupled assay if it shifts the equilibrium position of the GDH reaction in the oxidative deamination direction. This would result in an inaccurate under-count of ammonia produced by GLS which could be misinterpreted as GLS inhibition. **C)** Mock GLS reactions with 1.1 mM ammonium ion (representing 7.5% conversion of 15 mM Gln by GLS) in GLS assay buffer were supplemented with glutamate at the concentrations used to measure glutamate inhibition of GLS (0, 0.41, 1.2, 3.7, 11, 33, 100 mM). The mock GLS reactions (10 µL) were added to the GDH assay mix (190 µL; containing 3.4 mM α-ketoglutarate and 0.23 mM NADH), incubated for 40 minutes, then the absorbance at 340 nm was measured. The data were normalized to 0 mM glutamate as 100% activity and 0 mM ammonia as 0% activity. The data show the highest glutamate concentration (100 mM) added to the GLS reaction caused a small (~ 5%) under-count of ammonia detection by the GDH coupled assay, which is well above the IC_50_ for glutamate inhibition of WT GLS (5 mM).

**Table S1.** Crystallographic Data Collection and Model Refinement Statistics

| **Data Collection**  Space group P21  Cell dimensions  a (Å) 50.85  b (Å) 138.21  c (Å) 177.12  β (°) 93.58  Resolution (Å) 50.0 - 3.0  Unique reflections 49294  (2404)  Redundancy 6.9 (7.1)  Completeness (%) 100.0 (100.0)  CC1/2 0.99 (0.52) |
| --- |
| **Refinement**  Resolution (Å) 50 - 3.0  R_work_/R_free_ 0.19/0.21  RMSD  Bond length (Å) 0.01  Angle (°) 1.08  Ramachandran statistics  Favored regions (%) 94.96  Allowed regions (%) 4.46  Outliers (%) 0.57  Avg B-factors (Å^2^) 71.93  PDB ID 9PIA |
